# Biomedical event extraction from abstracts and full papers using search-based structured prediction

**DOI:** 10.1186/1471-2105-13-S11-S5

**Published:** 2012-06-26

**Authors:** Andreas Vlachos, Mark Craven

**Affiliations:** 1 Computer Laboratory, University of Cambridge, UK; 2 Department of Biostatistics and Medical Informatics, University of Wisconsin-Madison, USA

## Abstract

**Background:**

Biomedical event extraction has attracted substantial attention as it can assist researchers in understanding the plethora of interactions among genes that are described in publications in molecular biology. While most recent work has focused on abstracts, the BioNLP 2011 shared task evaluated the submitted systems on both abstracts and full papers. In this article, we describe our submission to the shared task which decomposes event extraction into a set of classification tasks that can be learned either independently or jointly using the *search-based structured prediction *framework. Our intention is to explore how these two learning paradigms compare in the context of the shared task.

**Results:**

We report that models learned using search-based structured prediction exceed the accuracy of independently learned classifiers by 8.3 points in F-score, with the gains being more pronounced on the more complex *Regulation *events (13.23 points). Furthermore, we show how the trade-off between recall and precision can be adjusted in both learning paradigms and that search-based structured prediction achieves better recall at all precision points. Finally, we report on experiments with a simple domain-adaptation method, resulting in the second-best performance achieved by a single system.

**Conclusions:**

We demonstrate that joint inference using the search-based structured prediction framework can achieve better performance than independently learned classifiers, thus demonstrating the potential of this learning paradigm for event extraction and other similarly complex information-extraction tasks.

## Background

The term *biomedical event extraction *is used to refer to the task of extracting descriptions of actions and relations among one or more entities from the biomedical literature. Since the scientific literature contains a wealth of information about relationships among gene products that is not contained in structured databases, there has been sustained interest in developing methods that are able to automatically extract these relationships of interest. In recent years, there have been two community-wide shared tasks focused on the semantically rich problem of event extraction. The 2009 and 2011 BioNLP shared tasks [1,2] involved extracting events composed from a handful of different relation types of varying complexity. In this article, we describe our submission to BioNLP 2011 shared task GENIA Task1 (BioNLP11ST-GE1) [3] and report on additional experiments we have conducted.

In our approach, we decompose event extraction into a set of classification tasks that can be learned either independently or jointly using the search-based structured prediction framework (SEARN) [4] in a formulation we proposed in earlier work [5]. SEARN is an algorithm that converts the problem of learning a model for structured prediction into learning a set of models for cost-sensitive classification (CSC). CSC is a task in which each training instance has a vector of misclassification costs associated with it, thus rendering some mistakes to be more expensive than others [6]. Compared to independently learned classifiers, SEARN is able to achieve better performance because its models are learned jointly. Thus, each of these models is able to incorporate features representing predictions made by the other ones, while taking into account possible mistakes made. Our intention is to explore how these two learning paradigms compare with each other, as well as with other approaches in the context of BioNLP11ST-GE1. In addition to reporting results using the official evaluation using F-score, we also explore the range of precision and recall points that are achievable by the two approaches. Moreover, we demonstrate how we can adjust the trade-off between recall and precision under SEARN by using a weighted loss function. Finally, we report on experiments with the simple domain adaptation method proposed by Daumé III [7], which creates a version of each feature for each domain.

An early shared task in biomedical information extraction was the Learning Language in Logic 2005 (LLL 2005) Genic interaction shared task [8], which focused on protein-protein interactions (PPI). However, the datasets involved were rather small in size, not allowing confident conclusions on system performances. LLL 2005 was followed by the protein-protein interaction pair subtask of BioCreative II [9]. In this subtask, the annotated datasets provided were produced by gathering curated interactions from relevant databases. This meant that there was no text-bound annotation, thus making the application and evaluation of existing NLP techniques difficult, resulting in rather low performances. Indicatively, the best performance achieved was 29 in F-score, while many of the teams scored below 10. More recently, the BioNLP 2009 shared task (BioNLP09ST) on event extraction [1] focused on a number of relations of varying complexity using a text-bound annotation scheme. The performances achieved ranged from 16 to 52 in F-score, suggesting improvements in task definitions, data annotation and participating systems. Following BioNLP09ST, the BioNLP 2011 shared task GE-NIA Task1 (BioNLP11ST-GE1) [3] used the same event extraction task definition as it predecessor, but evaluated the submitted systems on event extraction from both abstracts and full papers. It had 15 participants with performances ranging from 11.80 to 56.04 in F-score.

## Methods

### Event extraction decomposition

**Figure 1:**
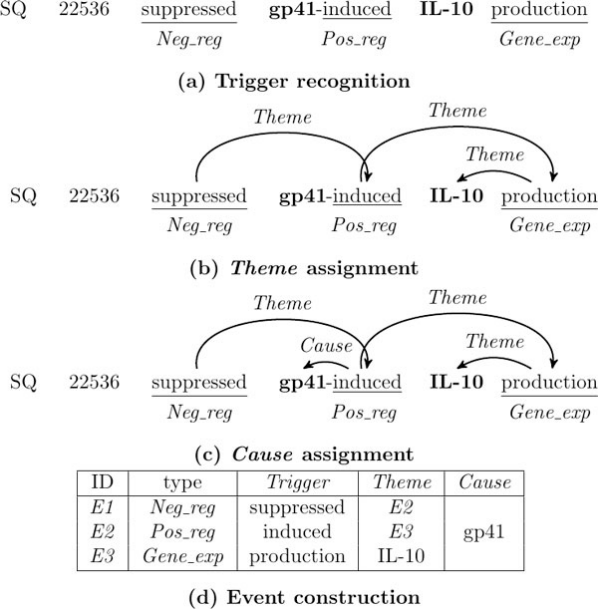
**The stages of our biomedical event extraction system**.

Each event consists of a *trigger *and one or more *arguments*. Nine event types are defined which can be grouped in three categories, namely *Simple*, *Binding *and *Regulation*. *Simple *events include *Gene*_*expression*, *Transcription*, *Protein*_*catabolism*, *Phosphorylation*, and *Localization *events. These have only one *Theme *argument which is a protein. *Binding *events have one or more protein *Themes*. *Regulation *events include *Positive*_*regulation*, *Negative*_*regulation *and *Regulation *and are the most complex ones as they have one obligatory *Theme *and one optional *Cause*, each of which can be either a protein or another event, thus resulting in nested events. The protein names are annotated in advance and any token in a sentence can be a trigger for one of the nine event types considered. Thus, the task can be viewed as a structured prediction problem in which the output for a given instance is a (possibly disconnected) directed acyclic graph (not necessarily a tree) in which vertices correspond to triggers or protein arguments, and edges represent relations between them. In an example demonstrating the complexity of the task, given the passage ". . . SQ 22536 suppressed **gp41**-induced **IL-10 **production in monocytes", systems should extract the three nested events shown in Figure 1.

Figure 1 describes the event extraction pipeline that is used throughout the paper. We assume that the sentences to be processed are parsed into syntactic dependencies and lemmatized. Each stage of the pipeline has its own module to perform the classification task needed, which is either a learned classifier (trigger recognition, *Theme*/*Cause *assignment) or a rule-based component (event construction).

#### Trigger recognition

In trigger recognition, the system decides whether a token acts as a trigger for one of the nine event types or not. Thus it is a 10-way classification task. We only consider tokens that are tagged as nouns, verbs or adjectives by the parser, as they cover the majority of the triggers in the data. This task is similar to word sense disambiguation, but it is simpler due to the restricted domain. The main features used in the classifier represent the lemma of the token which is sufficient to predict the event type correctly in most cases. In addition, we include features that conjoin each lemma with its part-of-speech tag. This allows us to handle words with the same nominal and verbal form that have different meanings, such as "lead". While the domain restricts most lemmas to one event type, there are some whose event type is determined by the context, e.g. "regulation" on its own denotes a *Regulation *event but in "positive regulation" it denotes a *Positive*_*regulation *event instead. In order to capture this phenomenon, we add as features the conjunction of each lemma with the lemma of the tokens immediately surrounding it, as well as with the lemmas of the tokens it has syntactic dependencies with.

#### Theme and Cause assignment

In *Theme *assignment, we form an agenda of candidate trigger-argument pairs for all trigger-protein combinations in the sentence and classify them as *Themes *or not. For each trigger-argument pair, a binary classifier is used to determine whether it has a *Theme *relation or not. Whenever a trigger is predicted to have a *Theme *argument, we form candidate pairs between all the *Regulation *triggers in the sentence and that trigger as the argument, thus allowing the prediction of nested events. Also, we remove candidate pairs that could result in directed cycles, as they are not allowed by the task.

The features used to predict whether a trigger-argument pair should be classified as a *Theme *are extracted from the syntactic dependency path and the textual string between them. In particular, we extract the shortest unlexicalized dependency path connecting each trigger-argument pair using Dijkstra's algorithm, allowing the paths to follow either dependency direction. One set of features represent these paths and in addition we have sets of features representing each path conjoined with the lemma, the PoS tag and the event type of the trigger, the type of the argument and the first and last lemmas in the dependency path. The latter help by providing some mild lexicalization. We also add features representing the textual string between the trigger and the argument, combined with the event type of the trigger. While not as informative as dependency paths, such features help in sentences where the parse is incorrect, as triggers and their arguments tend to appear near each other.

In *Cause *assignment, we form an agenda of candidate trigger-argument between the *Regulation *class triggers that were assigned at least one *Theme *and the protein names and the other triggers that were assigned a *Theme*. For each trigger-argument pair, a binary classifier is used to determine whether it has a *Cause *relation or not. We extract features as in *Theme *assignment, adding additional features representing the conjunction of the dependency path of the candidate pair with the path(s) from the trigger to its *Theme(s)*.

#### Event construction

In the event construction stage, we convert the predictions of the previous stages into events. If a *Binding *trigger is assigned multiple *Themes*, we choose to form either one event per *Theme *or one event with multiple *Themes*. For this purpose, we group the arguments of each nominal *Binding *trigger according to the first label in their dependency path and generate events using the cross-product of these groups. For example, assuming the parse was correct and all the *Themes *recognized, "interactions of **A **and **B **with **C**" would result in two *Binding *events with two *Themes *each, **A **with **C**, and **B **with **C **respectively. We add the exceptions that if two *Themes *are part of the same token (e.g. "**A**/**B **interactions"), or the trigger and one of the *Themes *are part of the same token, or the lemma of the trigger is "bind" then they form one *Binding *event with two *Themes*.

Finally, there are certain tokens such as "overexpress" that are consistently annotated with a *Simple *event type and a *Regulation *event type with the latter forming an event with the former as its *Theme*. In the event extraction decomposition used, we predict one event type per token so it is not possible to produce this event structure. Therefore, for the lemmas that have these additional *Regulation *events, we generate them heuristically using a dictionary.

### Structured prediction with SEARN

SEARN [4] forms the prediction of an instance *s *as a sequence of *T *multiclass predictions ${ŷ}_{1:T}$ made by a hypothesis *h*. The hypothesis consists of a set of classifiers that are learned jointly. Each prediction ${ŷ}_{t}$ can use features from *s *as well as from all the previous predictions ${ŷ}_{1:t-1}$. These multiclass predictions are referred to as *actions *and we adopt this term in order to distinguish them from the structured output prediction of an instance. The number of actions taken for an instance is not defined in advance but it is determined as the prediction is formed.

The SEARN algorithm is presented in Alg. 1. It initializes hypothesis *h *to the *optimal policy π *(step 2) which predicts the optimal action in each timestep *t *according to the gold standard. The optimal action at timestep *t *is the one that minimizes the overall loss over *s *assuming that all future actions ${ŷ}_{t+1:T}$ are also made optimally. The loss function is defined by the structured prediction task considered. Each iteration begins by making predictions for all instances *s *in the training data  (step 6). For each *s *and each action ${ŷ}_{t}$, a cost-sensitive classification (CSC) example is generated (steps 8-12). The features are extracted from *s *and the previous actions ${ŷ}_{1:t-1}$ (step 8). The cost for each possible action $y_{t}^{i}$ is estimated by predicting the remaining actions $y_{t+1:T}^{\prime }$ in *s *using *h *(step 10) and evaluating the cost incurred given that action (step 11). Using a CSC learning algorithm, a new hypothesis is learned (step 13) which is combined with the current one according to the interpolation parameter *β *(step 14). In order to interpolate between the learned hypotheses and the optimal policy we draw a random number between 0 and 1. If it is less than (1-*β*)*^iteration^*, then we use the optimal policy. Otherwise, we use the learned hypotheses which is a weighted ensemble of the hypotheses learned in each iteration (*h_new_*). The weights are set according to the equation in step 13, which results in hypotheses learned in earlier rounds becoming less important their more recent counterparts.

In each iteration, SEARN moves away from the optimal policy and instead uses the learned hypotheses when predicting (steps 6 and 10). Thus, each *h_new _*is adapted to the actions chosen by *h *instead of those of the optimal policy. When the dependence on the latter becomes insignificant (i.e. the probability of using the optimal policy becomes very small), the algorithm terminates and returns the weighted ensemble of learned hypotheses without the optimal policy.

The interpolation parameter *β *determines how fast SEARN moves away from the optimal policy, and as a result, how many iterations will be needed to minimize the dependence on it. Dependence in this context refers to the probability of using the optimal policy instead of the learned hypothesis in choosing an action during prediction. Conversely, in each iteration, the features extracted (Φ*_t _*in step 8) become progressively dependent on the actions chosen by the learned hypotheses instead of those of the optimal policy.

The decomposition of structured prediction into actions implies a search order. For some tasks such as part-of-speech (PoS) tagging, there is a natural left-to-right order in which the tokens are treated. However for others, including the task tackled in this paper, this ordering might not be appropriate. We discuss this issue in the next section.

Structural information under SEARN is incorporated in two ways. First, via the costs that are estimated using the loss over the instance rather than isolated actions, e.g. counting how many incorrect PoS tags will occur in the sentence if a given token is tagged as noun. Second, via the features extracted from the previous actions, e.g. the PoS tag predicted for the previous token can be a feature. Note that such features would be possible in a standard pipeline as well, but during training they would have to be extracted using the gold standard instead of the actual predictions made by the classifiers, as they would be extracted during testing. While it is possible to use cross-validation to train a pipeline on its own predictions, however this is rarely done in practice.

Finally, SEARN can be adapted to learn a pipeline of independently trained classifiers. To achieve this, *β *must be set to 1 so that there is only one iteration, the features that are dependent on previous actions must be removed, and the cost for each action must be set to 0 if it follows from the gold standard, or to 1 otherwise. This adaptation allows for a fair comparison between SEARN and a pipeline of independently learned classifiers.

### Biomedical event extraction with SEARN

In this section we describe how we use SEARN to learn the event extraction decomposition described earlier. Each instance is a sentence and the hypothesis learned in each iteration consists of a classifier for each stage of the pipeline, excluding event construction which is rule-based. For this purpose we need to concretely define the way the prediction of a structured instance is performed (step 6 in Alg. 1), the optimal policy, and the method used to estimate the cost for each action (steps 9-11 in Alg. 1).

**Algorithm 1 **SEARN

1: **Input: **labeled instances , *optimal policy π*, CSC learning algorithm *CSCL*, loss function ℓ

2: current policy *h *= *π*

3: **while ***h *depends significantly on *π ***do**

4:    Examples *E *= ∅

5:    **for ***s ***in ***S ***do**

6:       Predict $h\left(s\right)={ŷ}_{1}...{ŷ}_{T}$

7:       **for ${ŷ}_{t}$ in ***h*(*s*) **do**

8:          Extract features $\Phi_{t}=f\left(s,{ŷ}_{1:t-1}\right)$

9:          **for each **possible action $y_{t}^{i}$**do**

10:             Predict $y_{t+1:T}^{\prime }=h\left(s|{ŷ}_{1:t-1},y_{t}^{i}\right)$

11:             Estimate $c_{t}^{i}=\ell \left({ŷ}_{1:t-1},y_{t}^{i},y_{t+1:T}^{\prime }\right)$

12:       Add (Φ*_t_*, *c_t_*) to *E*

13:    Learn a classifier *h_new _*= *CSCL*(*E*)

14:    *h *= *βh_new _*+ (1 - *β*)*h*

15: **Output: **policy *h *without *π*

SEARN allows us to extract structural features for each action from the previous ones. During trigger recognition, we add as features the combination of the lemma of the token being classified and the event types (if any) assigned to the previous and the next token, as well as the event type assigned to the tokens that have syntactic dependencies with the token being classified. During *Theme *assignment, when considering a trigger-argument pair, we add features based on whether the pair forms an undirected cycle with previously predicted *Themes *(undirected *Theme *cycles are allowed in the task definition but they are relatively rare), whether the trigger has been assigned a protein as a *Theme *and the candidate *Theme *is an event trigger (and the reverse), and whether the argument is the *Theme *of a trigger with the same event type. We also add a feature indicating whether the trigger has three *Themes *predicted already, as triggers with more *Themes *are rare. During *Cause *assignment, we add features representing whether the trigger has been assigned a protein as a *Cause *and whether the candidate *Cause *is an event trigger.

Since the features extracted for an action depend on previous ones, we need to define a prediction order for the actions. Ideally, the actions predicted earlier should be less dependent on structural features and/or easier so that they can inform the more structure dependent/harder ones. In trigger recognition, we process the tokens from left to right since modifiers appearing before nouns tend to affect the meaning of the latter, e.g. "binding activity". In *Theme *and *Cause *assignment, we predict trigger-argument pairs in order of increasing dependency path length, assuming that, since they are the main source of features in these stages and shorter paths are less sparse, pairs containing shorter ones should be predicted more reliably. Trigger-argument pairs with the same dependency path length are predicted according to the order they were added to the agenda, i.e. pairs with proteins as arguments are predicted before those that have other triggers as arguments. While we found this ordering of the actions to work well in practice, it could be improved by taking into account other properties of the trigger-argument, e.g. how frequently we encountered its dependency path in the training data.

The loss function sums the number of false positive and false negative events, which is the evaluation measure of the shared task. The optimal policy is derived from the gold standard and returns the action that minimizes the loss over the sentence given the previous actions and assuming that all future actions are optimal. In trigger recognition it returns either the event type for tokens that are triggers or a "No trigger" label otherwise. In *Theme *assignment, for a given trigger-argument pair the optimal policy returns *Theme *only if the trigger is recognized correctly and the argument is indeed a *Theme *for that trigger according to the gold standard. In case the argument is another event, we require that its *Themes *have been recognized correctly as well. In *Cause *assignment, the requirements are the same as those for the *Themes*, but we also require that at least one *Theme *of the trigger in the trigger-argument pair to be considered correct. Consequently, if a trigger is predicted with the wrong event type, the optimal policy would not assign any *Themes *to it in order to avoid the false positive event it would incur. These additional checks are imposed by the task definition, under which events must have all their elements identified correctly. While the could reduce recall as the optimal policy avoids predicting some *Theme *edges, it allows the algorithm to learn how to minimize the losses incurred due to its own wrong decisions.

#### Cost estimation

**Figure 2:**
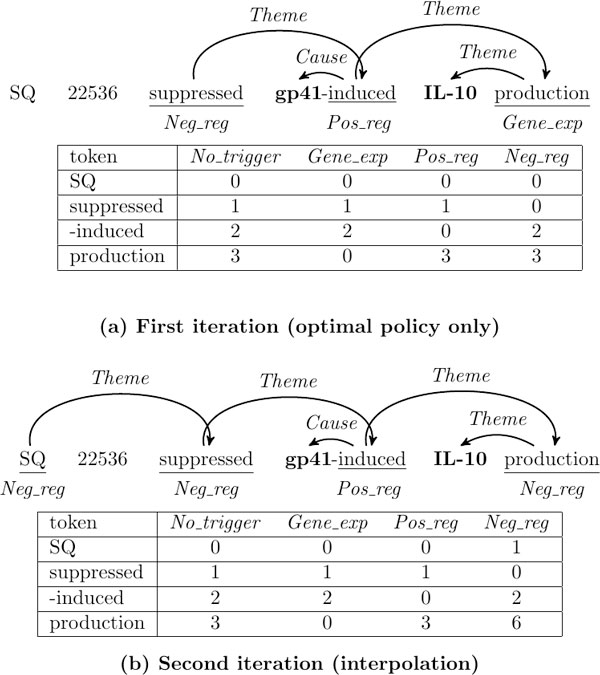
**Prediction (top of each panel) and cost sensitive examples for trigger recognition actions (bottom of each panel) in the first two SEARN iterations**.

Cost estimation (steps 6-12 in Alg. 1) is crucial to the successful application of SEARN. In order to highlight its importance, consider the example of Figure 2 focusing on trigger recognition.

In the first iteration (Figure 2(a)), the actions for the sentence will be made using the optimal policy only, thus replicating the gold standard. During costing, if a token is not a trigger according to the gold standard (e.g. "SQ"), then the cost for all actions is 0, as the optimal policy will not assign *Themes *to a trigger with incorrect event type. Such instances are ignored by the cost-sensitive learner.

In the second iteration (Figure 2(b)), the optimal policy is interpolated with the learned hypothesis, thus some of the actions are likely to be incorrect. Assume that "SQ" is incorrectly predicted to be a *Neg*_*reg *trigger and assigned a *Theme*. During costing, the action of labeling "SQ" as *Neg*_*reg *has a cost of 1, as it would result in a false positive event. Thus the learned hypothesis will be informed that it should not label "SQ" as a trigger as it would assign *Themes *to it incorrectly and it is adapted to handle its own mistakes. Note that the costs for the other actions for that token remain 0, assuming that the learned hypothesis would not assign *Themes *to "SQ" if it is predicted to be *Gene*_*exp*, *Pos*_*reg *or *No*_*trigger*. Similarly, the action of labeling "production" as *Neg*_*reg *in this iteration would incur a cost of 6, as the learned hypothesis would assign a *Theme *incorrectly, thus resulting in 3 false negative and 3 false positive events. Therefore, the learned hypothesis will be informed that assigning the wrong event type to "production" is worse than not predicting a trigger.

The interpolation between the optimal policy and the learned hypothesis is stochastic, i.e. in each iteration beyond the first one the actions are taken either by the optimal policy or by the learned hypotheses, as described in the section "Structured prediction with SEARN". Therefore, the cost estimates obtained in steps 10 and 11 of Alg. 1 vary according to the mistakes made by the learned hypothesis, thus affecting the cost estimates obtained. In order to obtain more reliable estimates, one can average over multiple samples for each action by repeating steps 10 and 11 of Alg. 1. However, the computational cost is effectively multiplied by the number of samples.

A different approach proposed by Daumé III [4] is to assume that all actions following the one we are costing are going to be optimal and use the optimal policy to approximate the prediction of the learned hypothesis in step 10 of Alg. 1. In tasks where the learned hypothesis is accurate enough, this has no performance loss and it is computationally efficient as the optimal policy is deterministic. However, in event extraction, the learned hypothesis is likely to make mistakes, thus the optimal policy would not provide a good approximation to it. In the example of Figure 2, this approach would not alter the costs between the two iterations, as the optimal policy would avoid assigning Themes to incorrectly recognized triggers, thus the learned hypothesis would not be informed of its mistakes.

In step 11 of Alg. 1, the cost of each action is estimated over the whole sentence. While this allows us to take structure into account, it can result in costs being affected by a part of the output that is not related to that action. This is likely to occur in event extraction, as sentences can often be long (more than 100 tokens) and contain disconnected event components in their output graphs. For this reason we use *focused costing *[5], in which the cost estimation for an action takes into account only the part of the output graph connected with that action. For example, in Figure 2 the cost estimation for "SQ" will ignore the events in the first iteration, while it will take them into account in the second one. Seen differently, *focused costing *results in more reliable cost estimates than the standard costing (for a given number of samples) by reducing the number of actions taken into account.

#### CSC learning with passive-aggressive algorithms

The SEARN framework requires a multiclass CSC algorithm to learn how to predict actions. This algorithm must be computationally fast during parameter learning and prediction, as in every iteration we need to learn a new hypothesis and to consider each possible action for each instance in order to construct the cost-sensitive examples.

Daumé III et al. [4] showed that it is possible to use any binary classification algorithm in order to perform multiclass CSC. This is achieved by reducing multiclass CSC to binary CSC using the weighted all-pairs algorithm [10] and in turn reducing CSC to binary classification using the costing algorithm [11]. The main drawback of this approach is that it relies on multiple subsamplings of the training data, which can be inefficient for large datasets and many classes. Zadrozny et al. [11] observed that it is more efficient to incorporate the costs in the loss of the classifier when possible. This can be relatively straightforward in binary problems, but not in the multiclass ones.

With these considerations in mind, we implement a multiclass CSC learning algorithm using the generalization of the online passive-aggressive (PA) algorithm for binary classification [12]. For each training example *x_t_*, the *K*-class linear classifier with *K *weight vectors $w_{t}^{\left(k\right)}$ makes a prediction ${ŷ}_{t}$ and suffers a loss ℓ*_t_*. In the case of multiclass CSC learning, each example has its own cost vector *c_t_*. If the loss is 0 then the weight vectors of the classifier are not updated (passive). Otherwise, the weight vectors are updated minimally so that the prediction on example *x_t _*is corrected (aggressive). The update takes into account the cost of the mistake and the aggressiveness parameter , which allows the algorithm to handle noisy data. Crammer et al. [12] describe three variants to perform the updates which differ in how the learning rate *τ_t _*is set for each example. In our experiments we used the variant named PA-II with prediction-based updates. Initial experiments showed little difference in accuracy between the variants, which is in agreement with the observations reported by Crammer et al.

The full algorithm is presented in Alg. 2. Since we are operating in a batch learning setting (i.e. we have access to all the training examples), we perform multiple rounds and average the weight vectors obtained, as in the averaged perceptron [13]. Furthermore, since online learning depends on the order of the training examples but our data does not have a temporal aspect, we shuffle the examples in the beginning of each round.

## Results

In this section we compare the event extraction accuracy achieved by the system based on independently learned classifiers (henceforth *independent*) versus the accuracy achieved by the system learning classifiers under SEARN. The purpose of these experiments is to assess the benefits of joint learning under SEARN. In the results reported below, we follow the dataset split of BioNLP11ST-GE1, namely 800 abstracts and five full articles for training, 150 abstracts and five full articles for development, and 260 abstracts and five full articles for testing. To put these results in a wider context, we also compare against the other systems that participated in BioNLP11ST-GE1.

For both *independent *and SEARN the aggressiveness parameter of PA and the number of rounds in parameter learning are set by tuning on 10% of the training set. For SEARN, we also set the interpolation parameter *β *to 0.3 and use 12 iterations. Thus, in the final iteration the probability of using the optimal policy is (1 - 0.3)^12 ^≈ 0.01. These parameters were tuned in preliminary experiments using the development data. For syntactic parsing, we use the output of the re-ranking parser [14] adapted to the biomedical domain [15], as provided by the shared task organizers in the Stanford collapsed dependencies with conjunct dependency propagation [16]. The use of this publicly available resource allows for easy replication of our experiments. Lemmatization is performed using *morpha *[17]. No other knowledge sources or tools are used. A pre-processing step we perform on the training data is to reduce the multi-token triggers in the gold standard to their syntactic heads. This procedure simplifies the task of assigning arguments to triggers and, as the evaluation variant used allows approximate trigger matching, it does not result in performance loss.

Table 1 reports the Recall/Precision/F-score achieved by *independent *and SEARN in each stage, as well as the overall performance on the development set. SEARN obtains better performance on the development set by 6.75 F-score points. The difference is more pronounced on the more complex *Regulation *events where SEARN achieves 41.36 versus 29.13. Table 2 contains detailed results per event type and class. Note that while the trigger classifier learned with SEARN overpredicts (its precision is 29.78), the *Theme *and *Cause *classifiers maintain relatively high precision with substantially higher recall as they are learned jointly with it. As triggers that do not form events are ignored by the evaluation, trigger overprediction without event overprediction does not result in performance loss.

**Table 1:** R(ecall)/P(recision)/F(-score) on the development dataset.

	*independent *(R/P/F)			SEARN (R/P/F)		
trigger	52.82	66.76	58.98	83.65	29.78	43.92
*Theme*	46.23	79.03	58.34	63.63	71.82	67.48
*Cause*	15.16	58.49	24.08	31.79	49.06	38.57

Event	35.68	69.39	47.12	49.15	59.60	53.87

Each row reports the results for each stage of the event extraction decomposition, with the last row containing the overall event extraction performance.

**Table 2:** Detailed results on the development data using independently learned classifiers and SEARN

		*independent*			SEARN	
**Event Type/Class**	**Recall**	**Precision**	**F-score**	**Recall**	**Precision**	**F-score**

*Gene*_*expression*	67.02	85.20	75.03	73.43	78.88	76.06
*Transcription*	34.81	90.16	50.23	48.73	70.00	57.46
*Protein*_*catabolism*	69.57	94.12	80.00	69.57	76.19	72.73
*Phosphorylation*	71.17	86.81	78.22	81.08	90.91	85.71
*Localization*	62.69	80.77	70.59	71.64	77.42	74.42

*Simple *(TOTAL)	62.64	85.66	72.36	70.49	78.95	74.48

*Binding*	28.42	63.10	39.19	40.21	62.24	48.86

*Simple+Binding *(TOTAL)	54.02	81.78	65.06	62.86	75.67	68.67

*Regulation*	14.04	44.57	21.35	32.19	37.90	34.81
*Positive*_*regulation*	22.02	55.00	31.45	40.24	46.42	43.11
*Negative*_*regulation*	20.38	48.73	28.74	35.46	50.61	41.70

*Regulation *(TOTAL)	20.26	51.81	29.13	37.63	45.91	41.36

TOTAL	35.68	69.39	47.12	49.15	59.60	53.87

**Algorithm 2 **Passive-aggressive CSC learning

1: **Input **: training examples $X=x_{1}...x_{T}$, cost vectors *c*_1 _. . . *c_T _*≥ 0, rounds *R*, aggressiveness 

2: **Initialize **weights $w_{0}^{\left(k\right)}=\left(0,...,0\right)$

3: **for ***r *= 1,..., *R ***do**

4:    **Shuffle **

5:    **for **$x_{t}\in X$**do**

6:          Predict ${ŷ}_{t}=argmax_{k}\left(w_{t}^{\left(k\right)}\cdot x_{t}\right)$

7:          Receive cost vector *c_t _*≥ 0

8:          **if **$c_{t}^{\left({ŷ}_{t}\right)}>0$**then**

9:          Suffer loss $\ell_{t}=w_{t}^{\left({ŷ}_{t}\right)}\cdot x_{t}-w_{t}^{\left(y_{t}\right)}\cdot x_{t}\sqrt{c_{t}^{\left({ŷ}_{t}\right)}}$

10:          Set learning rate $\tau_{t}=\frac{\ell_{t}}{||x_{t}|{|}^{2}+\frac{1}{2c}}$

11:          Update $w_{t+1}^{\left(y_{t}\right)}=w_{t}+\tau_{t}x_{t}$

12:          Update $w_{t+1}^{\left({ŷ}_{t}\right)}=w_{t}-\tau_{t}x_{t}$

13: **Average **$w_{avg}=\frac{1}{T\times R}\sum_{i=0}^{T\times R}w_{i}$

The results on the test dataset using SEARN are 46.67/61.63/53.12 (Recall/Precision/F-score) which would have ranked fourth in the shared task, 2.92 F-score points below the best performing ensemble system FAUST [18]. (These numbers are higher than the ones reported in the official results labeled UW-Madison due to fixing a bug.) On the same dataset, the *independent *system achieves 33.45/67.87/44.82, which while it would have ranked eighth in the shared task (out of a total of 15 participants), it is 8.3 F-score points below the result achieved with SEARN. In the full papers part of the corpus, our approach using SEARN would have ranked second with 52.98 F-score points, slightly below the best reported performance at 53.14 by UMass [19]. While a direct comparison between learning frameworks is difficult due to the differences in task decomposition and feature extraction, we hypothesize that the superior performance of these systems is partly due to learning how to construct *Binding *events, while our approach uses heuristics for this task. However, it is possible to model *Binding *event construction with a classifier and learn it jointly with SEARN, which we leave for future work.

### Evaluation at varying recall-precision curves

In the previous section we evaluated the accuracy of the event extraction systems discussed using F-score, which by default favors balanced precision and recall scores. While SEARN achieves a better F-score than *independent*, it is important to note that they operate at different precision levels, with *independent *being substantially more precise at 69.39% versus 59.60%. Therefore it is reasonable to ask whether SEARN achieves higher F-score simply because it operates at lower precision, thus if it was forced to operate at the same precision this would result in lower recall (and therefore F-score) than the one achieved by *independent*. In other words, the question we ask is whether SEARN learns more than a good set of thresholds for the classifiers used at each stage of the event extraction decomposition.

In this section, we explore the behavior of the two systems by adjusting the scores returned during prediction by the classifier used at each stage. In particular, we alter the score returned by the classifier for the negative class of each stage (*No*_*Trigger *for trigger recognition, *No*_*Theme *for *Theme *assignment and *No*_*Cause *for *Cause *assignment) by a parameter that can be either positive, thus resulting in over-generation, or negative, thus resulting in under-generation. Each stage has its own parameter, thus each experimental run is defined by a set of three parameter values. Altogether, we investigated 1,000 sets of parameter values for both systems, the results of which we evaluate on the development data.

**Figure 3:**
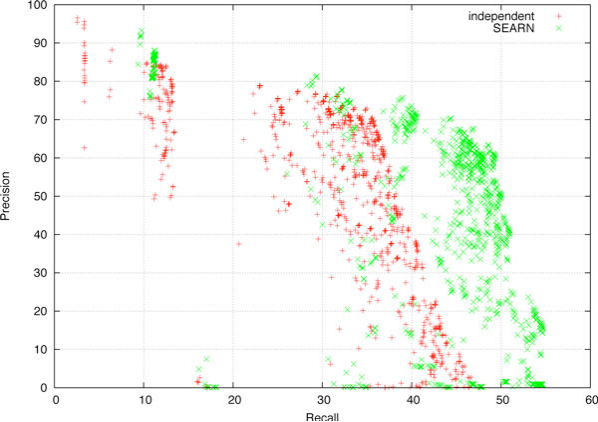
**Recall-precision points resulting from different parameter values for independent and SEARN**.

The results of Figure 3 demonstrate that SEARN achieves better recall than *independent *at all precision levels. In particular, at 69% precision (the precision of the *independent *system on this dataset reported in the previous section), SEARN achieves 44% recall versus 36%. This is also the case at even higher precision levels. For example, SEARN achieves 29% recall at 80% precision, compared to 13% by *independent*. Finally, these observations are confirmed at the other end of the precision-recall trade-off. For example, at 20% precision SEARN achieves 54% recall compared to 43% by *independent*. Thus we confirm that the improved predictive accuracy of SEARN is not only due to adjusting classification thresholds, but also due to generating appropriate training examples and learning structural feature weights.

### Controlling the trade-off between recall and precision

In the previous section we adjusted the trade-off between precision and recall in order to obtain a more complete comparison between classifiers learned with SEARN and classifiers learned independently. The ability to adjust this trade-off is of interest to users of event extraction systems, as they frequently need to adapt the behavior of a system to particular needs. For example, if a system is going to be used to populate a knowledge base whose users are not expected to verify its contents, then precision is more important than recall. Conversely, if it is going to be used by users to navigate through the biomedical literature and recover rarely mentioned facts about proteins, then recall is more important than precision. Similar observations were made in the context of biomedical named entity recognition by Carpenter [20].

As described earlier, the results reported in the previous section were obtained by evaluating the effect of 1,000 sets of parameter values to adjust the classification scores at each stage. It is important to note that the effect of these values is not straightforward to anticipate, as it is difficult to predict how the classifiers for each stage will interact with each other. For example, it is impossible to know in advance how to adjust the *Theme *assignment classifier if we adjust the trigger recognizer to over-generate. Furthermore, in the case of complex classification pipelines, the effect of each parameter can be hard to predict, e.g. while over-generating *Causes *is expected to increase recall since it results in more events predicted, it could also have the opposite effect, as it can change correctly extracted *Regulation *events that do not have such arguments into incorrect ones. These issues are visible in the results of Figure 3, where for each recall level there is a range of precision values obtained by each system, some of them well below the best one or even 0.

In order to choose the best set of parameter values at various precision levels, it is customary to use a development set, as done in order to report the results in the previous section. However this is not always desirable, as it requires part of the available annotated data to be withheld for this purpose and thus not used for training. Furthermore, the procedure to find the parameter values that result in the desired trade-off between recall and precision must be repeated each time there is a change in the event extraction system.

Instead of adjusting classification scores, it is possible under SEARN to adjust the trade-off between precision and recall via the loss function ℓ used to estimate the cost of each action (step 11 in Algorithm 1). In our approach, as described in the section "Biomedical event extraction with SEARN", the loss function is the sum of the numbers of false positives and false negatives. Therefore, in order to learn a system with higher precision, we multiply the number of false positives with a positive weight, and conversely, in order to learn a system with higher recall we multiply the number of false negatives with a positive weight.

**Figure 4:**
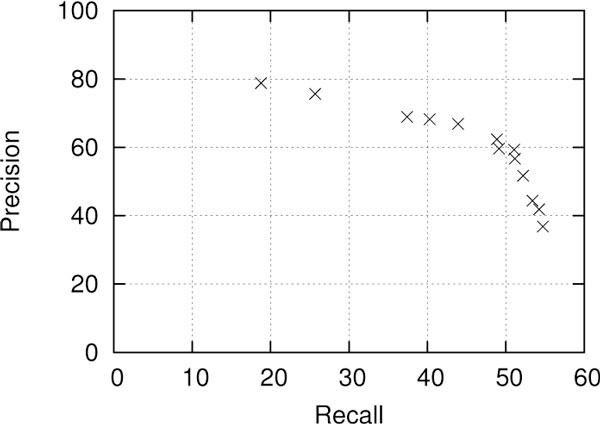
**Recall-precision points obtained for SEARN using different weights for false positives and false negatives**.

The results obtained using SEARN with different weights on false positives and false negatives are shown in Figure 4. In each experiment, one of these weights was kept to 1, while the other one was set to 2^1...6^, thus resulting in 12 experiments in total. It can be observed that in all cases the trade-off achieved is a reasonable one, i.e. favoring precision over recall never results in the latter becoming prohibitively low, as well as the reverse. This demonstrates that the classifiers learned jointly under SEARN are adapted to each other in order to adjust the balance between precision and recall. The benefits of this method are more pronounced at the higher recall levels, for example it obtains 54% recall at 41% precision, while the same recall was possible only at 18% precision in the previous section. Furthermore, while the trade-off achieved at high precision levels is not always as good as the one obtained by adjusting the scores of the classifiers directly, it is never substantially worse. Most importantly, using weights on false positives and false negatives in the loss function is very stable and thus it can be used without a development set.

### Experiments with domain adaptation

BioNLP11ST-GE1 evaluated event extraction on abstracts and full papers. While the annotation guidelines used were the same, full papers are likely to contain richer vocabulary and linguistic phenomena than the abstracts. Since we have annotated data for both we decided to address this task as a supervised domain adaptation problem. We experimented with the domain adaptation method proposed by Daumé III [7], which creates multiple versions for each feature by conjoining it with the domain label of the instance it is extracted from (abstracts or full papers). For example, during trigger recognition, the feature representing the lemma of a token becomes three features: the original lemma feature, a lemma feature for the abstracts domain and a lemma feature for the full papers domain. For each token, only two of these features will be active, according to the domain of the sentence the token is found in. For example, if the token is found in a sentence of an abstract, only the original lemma feature and the abstracts domain lemma feature will be active.

In our experiments, this simple domain adaptation method improved the accuracy of the classifiers trained under SEARN by 0.5 F-score points on the development and 0.41 F-score points on the test set, mainly by improving accuracy on the abstracts while preserving the already high accuracy on the full papers. This improvement is due to the domain-specific versions of the features that allow the flexibility to model the particularities of each domain independently. This version of the system would have ranked third overall with 53.53 F-score points, and second if we do not take the best-performing ensemble system FAUST [18] into consideration. Table 3 contains detailed results per event type, event class and domain. In the regulation events that are more difficult to extract it would have ranked third overall and in the regulation events of the full papers it would have ranked first with 42.45 F-score points, 1.89 points better than the best-performing ensemble system FAUST. We hypothesize that the relatively limited impact of domain adaptation is due to the sparse features used in the stages of the event extraction decomposition, which become even sparser using this domain adaptation method, thus rendering the learning of appropriate weights for them harder.

**Table 3:** Detailed results on the test data using SEARN with domain adaptation

Domain		abstracts+full		abstracts	full papers
**Event Type/Class**	**Recall**	**Precision**	**F-score**	**F-score**	**F-score**

*Gene*_*expression*	69.46	80.65	74.64	72.63	79.35
*Transcription*	44.83	62.40	52.17	53.11	48.28
*Protein*_*catabolism*	73.33	42.31	53.66	70.97	0.00
*Phosphorylation*	81.62	88.82	85.07	83.08	90.53
*Localization*	45.03	88.66	59.72	61.72	43.75

*Simple *(TOTAL)	65.22	79.78	71.77	70.32	75.80

*Binding*	38.09	57.36	45.78	46.67	43.72

*Simple+Binding *(TOTAL)	58.75	75.23	65.98	65.27	67.87

*Regulation*	32.99	41.50	36.76	38.13	32.77
*Positive*_*regulation*	40.82	51.62	45.59	44.69	47.36
*Negative*_*regulation*	38.35	43.89	40.93	43.80	35.64

*Regulation *(TOTAL)	38.97	48.05	43.04	43.33	42.45

TOTAL	48.10	60.34	53.53	53.79	52.93

## Conclusions

We presented a joint inference approach to the BioNLP11ST-GE1 event extraction task using SEARN which converts a structured prediction task into a set of CSC tasks whose models are learned jointly. Our results demonstrate that SEARN achieves substantial performance gains over independently learned classifiers using the same features at all precision levels. Furthermore, we suggested an efficient method to adjust the trade-off between recall and precision under SEARN in order to accommodate different usage scenarios. Finally, we were able to improve our performance further using a simple domain adaptation method in order to handle the differences between abstracts and full papers. In the course of our experiments, we reported the second-best event extraction results by a single system.

## Competing interests

The authors declare that they have no competing interests.

## Authors' contributions

AV wrote the code and ran the experiments. Both authors were involved in designing the approach and writing the manuscript.
